# A label-free optical system with a nanohole array biosensor for discriminating live single cancer cells from normal cells

**DOI:** 10.1515/nanoph-2021-0499

**Published:** 2021-12-03

**Authors:** Alfredo Franco, Verónica Vidal, Marcos Gómez, Olga Gutiérrez, María Martino, Francisco González, Fernando Moreno, José L. Fernández-Luna

**Affiliations:** Department of Applied Physics, Faculty of Sciences, University of Cantabria, Santander 39013, Spain; Genetics Unit, Valdecilla University Hospital, Santander 39008, Spain; Department of Surgery, Valdecilla University Hospital, Santander 39008, Spain; Department of Pathology, Valdecilla University Hospital, Santander 39008, Spain

**Keywords:** actin cortex, cancer cell, extraordinary optical transmission, plasmonics

## Abstract

Developing a simple, fast, and label-free method for discrimination between live cancer cells and normal cells in biological samples still remains a challenge. Here, a system is described that fulfills these features to analyze individual living cells. The system consists of a gold nanohole array biosensor plus a microscope optical design to isolate the spectral response of a single cell. It is demonstrated that differences in the spectral behavior between tumor (colorectal cancer cell lines and primary cells from colorectal cancer tissue) and non-tumor cells (peripheral blood mononuclear cells, skin fibroblasts and colon epithelial cells) are influenced by the actin cortex, which lies within the short penetration depth of the surface plasmon electromagnetic field. The efficacy of this system was assessed by the analysis of about one thousand single cells showing the highest discrimination capacity between normal colon epithelial cells and colorectal cancer cells from surgical specimens, with values of sensitivity and specificity ranging 80–100% and 87–100%, respectively. It is also demonstrated that cell discrimination capacity of the system is highly reduced by disrupting the formation of actin cortex. This plasmonic system may find wide applications in biomedicine and to study key cellular processes that involve the actin cortex, including proliferation, differentiation, and migration.

## 1 Introduction

Discovering the extraordinary optical transmission (EOT) phenomenon [1] has revolutionized sensing techniques, particularly those of biomedical interest, due to its high sensitivity [2, 3]. Different lab-on-a-chip sensing tools for detecting small quantities of biomolecules dispersed in fluids have been developed [4, 5]. Their common sensing feature is the detection of changes in the optical properties, refractive index (RI), of the material in contact with the metallic surface as a proof of its physical/chemical changes. The excited surface plasmons in nanostructured metallic films carry an electromagnetic energy “skin” through evanescent waves, hundreds of nanometers thick, whose interaction with biomatter “touching” the metallic interface can reveal subtle changes in its physical/chemical features. These changes manifest by modification of the spectral properties (position/width) of the transmitted intensity through the nanostructured surface. Potential biomedical applications of plasmonics are widely described [6], [7], [8], [9]. EOT-based biosensors have been extensively used to detect DNA molecules and protein conformational changes, evaluate the binding kinetics and affinity of small molecules to their target proteins in drug screening studies and identify cellular responses to microenvironmental factors [10, 11]. However, its application to detect big and highly complex biological structures, such as cells, has been barely explored. Most studies have focused on cell–substrate interactions, including characterization of cell shape on gold films [10] and assessing the cell–substrate adhesion strength [12] or cellular kinetics by EOT [13]. It has been anticipated that integrating EOT with scanning ion-conductance microscopy and atomic force microscopy could strengthen its capability to analyze single cells [10]. Plasmonic nanohole biosensors functionalized with specific antibodies and EOT have been recently described for detection of bacterial infections in biological fluids [14, 15]. There have also been described systems that use nanostructured array biosensors for analyses of tumor cells, which may detect secretion of cellular metabolites through either electrochemical sensing [16] or EOT [17], but none of the currently existing systems can discriminate between tumor and non-tumor cells without labeling cells or functionalizing the nanostructured surface.

Cell identification and biological processes can be revealed through changes in the optical properties of cells and consequently, their RI is a relevant biophysical parameter [18, 19]. In this sense, EOT-based biosensors could represent a suitable tool for detecting changes in these properties and, for instance, discriminating between tumor and non-tumor cells. Most of the label-free approaches based on EOT are not aimed at studying single cells. Also, it is important to remark that although EOT-based biosensing techniques do not show the highest sensitivity compared with those based on propagating surface plasmons (including imaging techniques), they remain an attractive platform for biosensing due to simple alignment and simple integration of the optical setup [20]. This is an important feature for implementation in healthcare settings. By considering that plasmon penetration depth is about 200 nm for gold nanostructured films [21] and that eukaryotic cells usually have sizes larger than 10 μm, EOT-based techniques would only sense the outer layer of the cell. This region includes the plasma membrane, composed of a phospholipid bilayer and glycoproteins, and a complex network of polymeric actin (F-actin) assembled into long filaments (actin cortex) attached to proteins, just underneath the plasma membrane. Actin cortex is necessary to allow cell shape changes in processes like cell division, cell migration and tumor cell invasion [22, 23] by means of an actin polymerization–depolymerization mechanism and generation of tensile forces [24] which makes this network structure an interesting biomarker to study cell dynamics. Tumorigenesis induces actin cortex remodeling, and tumor cell deformability is largely determined by myosin-driven cortical tension and actin fiber architecture at the cell cortex [25]. Difficulty to study these processes resides in the fact that actin cortex is highly heterogeneous in structure, molecular organization and dynamics. For instance, some studies describe a thickness of about 100 nm for mitotic cells and up to 500 nm for adherent cells [26, 27].

Here, we report the use of EOT to analyze single cancer and normal cells on a gold nanohole array. The spectral position of the transmitted light greatly depends on the RI of the material placed within the first 200 nm above the array, a region with the strongest plasmonic resonance, which makes this technique potentially suitable to study changes in structure and organization of the outer layer of the cell. We have studied differences in the spectral shift and transmittance between colorectal cancer cells (cell lines and primary cells) and different normal cell types (peripheral blood mononuclear cells (PBMCs), fibroblasts and epithelial cells) and have determined the contribution of membrane proteins and actin cortex. EOT-based biosensors pave the way for studies aiming at characterizing cancer cell dynamics and exploring novel biomedical applications.

## 2 Materials and methods

### 2.1 Optical system and experimental setup

**Figure 1:**
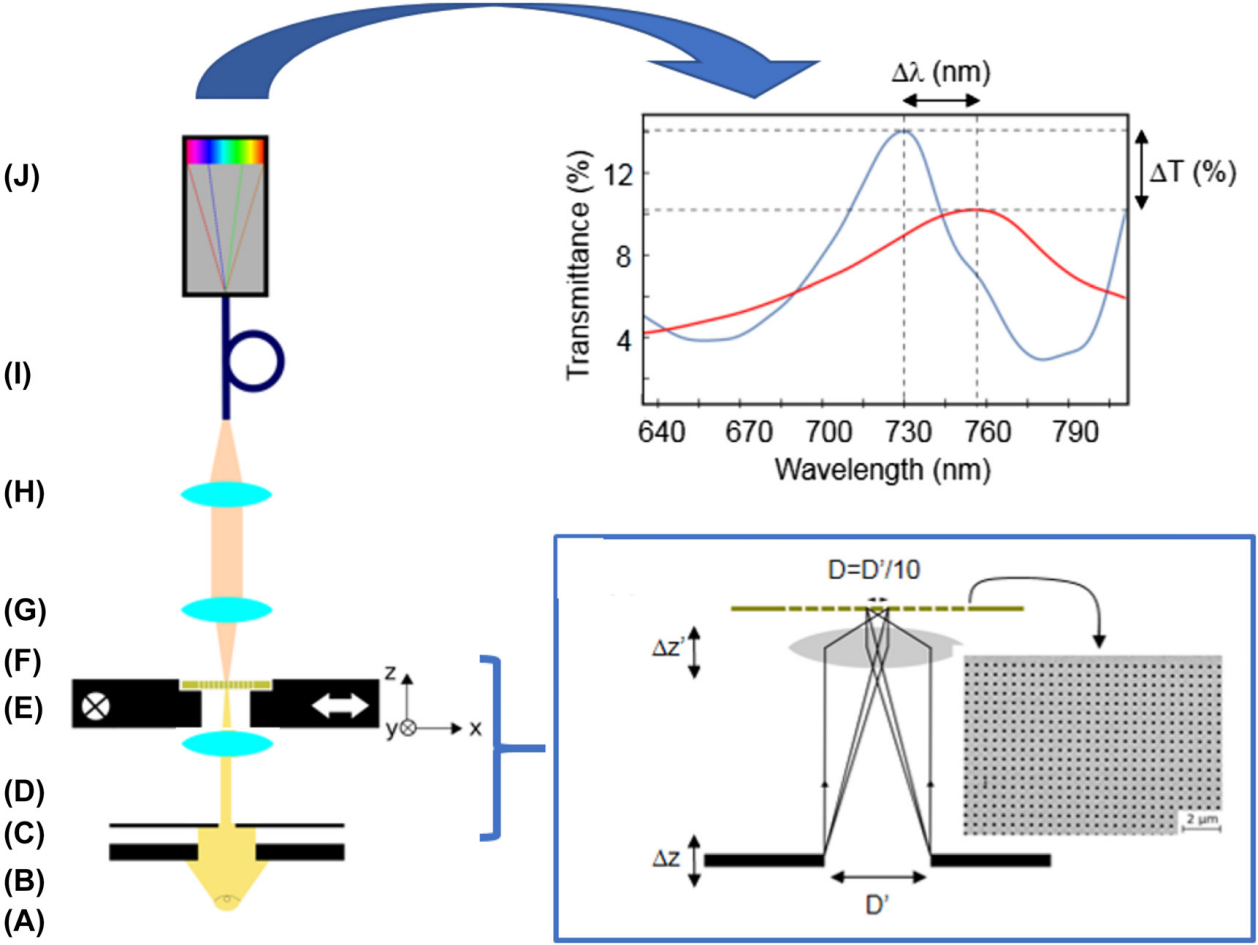
**Left**: Nanohole array sensor-based optical system proposed in this work. (A) The light source is a 100 W halogen lamp; (B) Field stop: the minimum field stop diameter is 1.5 mm; (C) Field pinhole: it is a geometric tailorable pinhole; (D) The condenser is an achromatic and aplanatic lens with 0.5 numerical aperture and object distance equal to 1.6 mm; (E) The sample-holder is a motorized stage for displacements as small as 100 nm along both *X* and *Y* directions. It is governed by means of a prior-Scan III controller; (F) The nanohole patterned sensor; (G) The objective lens is a 20× bright field lens; (H) The collecting lens is a 4.5 mm focal distance lens; (I) The optical fiber (Ocean Optics) has a 200 μm core, optimized for its use in the UV–visible spectral region; (J) Andor Shamrock spectrograph operating with a 300 μm entrance slit and coupled to an Andor IDUS CCD camera with an integration time of 0.1 s. **Top-right**: An example of the output of the spectrograph. Δ*λ* corresponds to the spectral shift when a cell is located on the biosensor area and Δ*T* to the change in the transmission. **Bottom-right**: Detail of the ensemble (C)–(E) where the condenser lens produces an image of the diaphragm (C) on the sensor chip with a magnification 1/10.

The system designed for cell analysis is a modified version of an optical upright Nikon-Eclipse Ni bright field transmission microscope (Figure 1) [28]. The system can measure the transmission spectrum in regions as small as few μm^2^. The diameter enclosing such regions is going to be denoted by *D* from now on. Besides, the device is also able to scan the surface and to measure the spectra of regions of diameter *D*, at a rate of 16 μm^2^/s. Results can be visualized as maps of the chip surface, where *D* × *D* μm^2^ pixels are associated to the maximum spectral wavelength in the restricted regions where the spectra were measured. In this setup, the pupil of the microscope built-in field stop is modified by means of the incorporation of a tailored pinhole (denoted as field pinhole from now on), placed at a distance Δ*z* above the field stop. The condenser lens is shifted to a new position placed at a distance Δ*z*′ above its built-in position in such a way that the new distances from the nanohole patterned surface to the condensing lens and to the field pinhole produces a 0.1 lateral magnification. Consequently, if the light passes through a circular field pinhole of diameter *D*′, it is focused on the nanopatterned surface in a circular spot of diameter *D* = *D*′/10. The light transmitted through the illuminated area of the chip surface is collected by the objective lens and, by means of the collecting lens, it is focused on the entrance of the optical fiber to be transmitted to the spectrograph where its spectrum is acquired. To increase the signal-noise ratio, the spectra are the result of the accumulation of 60 consecutive spectral measurements. Figure 1 summarizes all the optical setup features. In addition, the introduction of a field pinhole in the optical design opens the possibility to control the shape and size of the illumination spot. Restriction in the illumination area allows us to inspect locally the chip surface with an increased sensitivity because the transmission spectra are sensitive to local characteristics (optical, geometrical, etc.) of the illuminated region.

**Figure 2:**
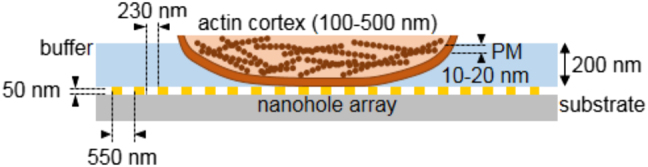
Scheme of the nanohole array configuration. Sensing chips are 50 nm thick gold films (99.999%) deposited on a glass slide coated with a 3 nm Ti film. The nanostructures are square periodic arrays of circular nanoholes. The period is 550 nm and the nanohole average diameter is 230 nm which are standard parameters to give a sensitivity of the order of 400 nm/RIU for a wavelength of 750 nm for the maximum transmitted intensity at the plasmon resonance. Penetration depth of localized surface plasmon is about 200 nm. The part of the cell that penetrates into the surface plasmon includes the plasma membrane (PM) (10–20 nm) and the actin cortex (100–500 nm).

Figure 2 shows a detail of the sensing area where the parameters characterizing the nanohole metallic film (period = 550 nm, hole diameter = 230 nm, film thickness = 50 nm and plasmon penetration depth ≈ 200 nm) on a glass substrate and the part of the cell under the plasmon influence are also indicated (thicknesses of the actin cortex = 100–500 nm and plasma membrane (PM) = 10–20 nm). For biosensing purposes, changes in the local effective RI in the illuminated volume close to the sensing chip surface depend on the optical properties and volume of any “imperfection” (biological or simply due to the manufacturing process [28]) with respect to an ideal sensing chip. Thus, a reduced illuminated area increases the fill factor due to that “imperfection” by modifying the effective RI of the volume close to the sensing surface [21].

### 2.2 Effective refractive index model

The measured spectra depend strongly on the effective RI (*n*_eff_) of the medium enclosed in the volume (*V*), determined by both the plasmon skin depth (200 nm) and the size of the illuminating spot, *D*, in such a way that *V* = 200*π*(*D*/2)^2^ nm^3^. As the effective RI increases, the transmitted spectra shift to longer wavelengths, according to the optical sensitivity (*S*) of the gold nanohole array. The latter is defined as the spectral wavelength shift ( Δλ ) per RI unit variation. Thus, Δλ relates to the RI of such part of the cell closest to the gold surface (*n*_cell_) in the following way [29].

(1) $$\Delta \lambda =S\left[\left(n_{\text{cell}}f_{\text{cell}}+n_{\text{buffer}}f_{\text{buffer}}\right)-n_{\text{buffer}}\right]\text{ ,}$$

where the expression in square brackets is the change in the effective RI (Δ*n*_eff_) due to the fraction of the volume (*V*) filled by a single cell ( $f_{\text{cell}}$ ), with respect to the RI of the buffer ( $n_{\text{buffer}}$ ), whose fill factor ( $f_{\text{buffer}}$ ) complements the one of the single cell, i.e. $f_{\text{cell}}+f_{\text{buffer}}=1$ .

**Figure 3:**
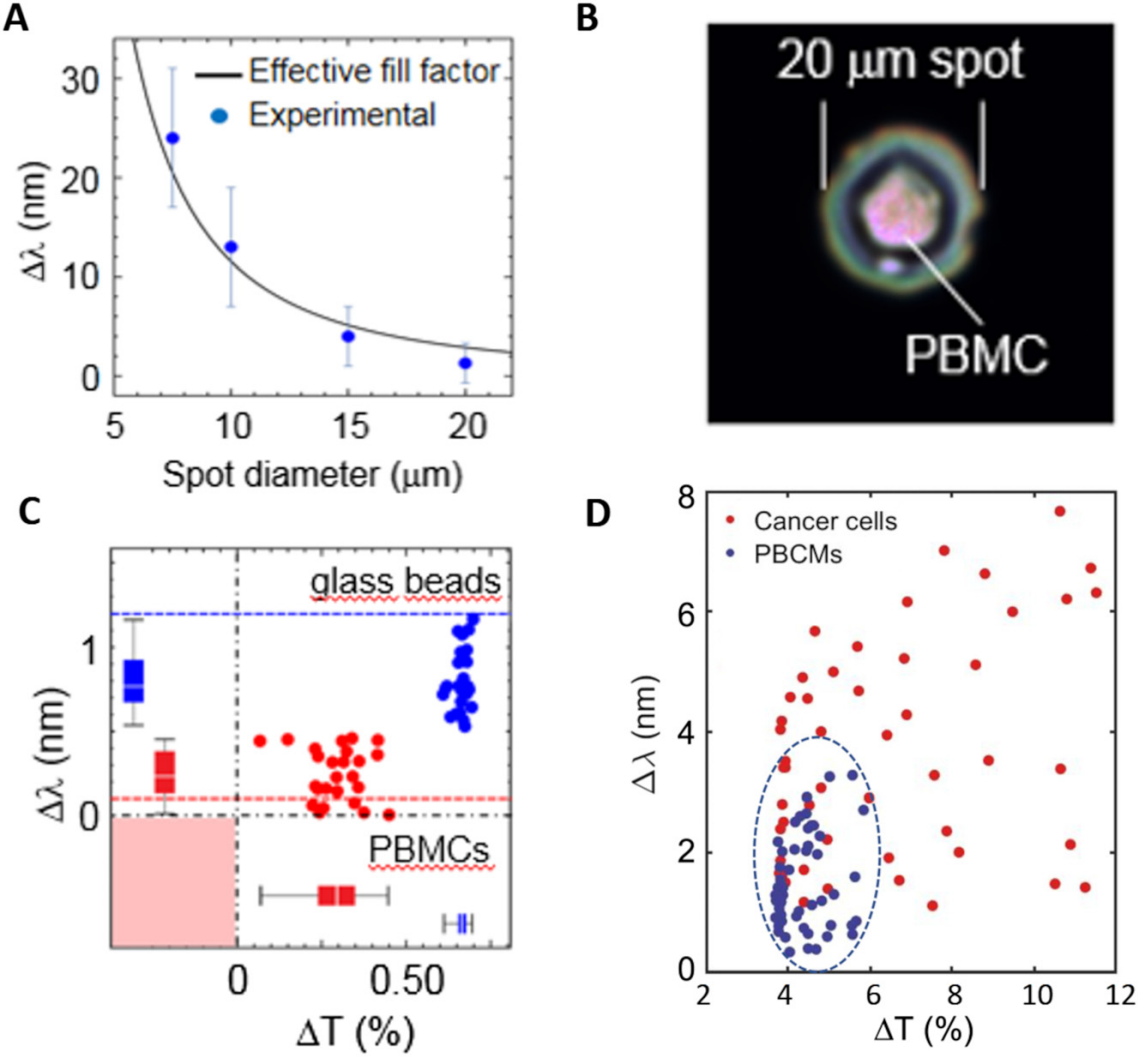
Effective refractive index model: (A) Spectral shift due to single glass microspheres as a function of the illumination spot size, D. Dots are experimental data and solid line represents a theoretical fit to the model described in the text. (B) A single PBMC on the nanohole array illuminated with a spot size of 20 μm. (C) Glass beads (10 μm in diameter) and PBMCs (70–90% lymphocytes) were analyzed with an illumination spot of 20 μm by determining the spectral shift and the change in transmittance. Dash-dotted lines mark the values of 0 for Δ*λ* and Δ*T*. Blue dotted line marks the spectral shift of the glass microbeads obtained according to the effective refractive index model described in Sect. 2.2. Red dotted line marks the spectral shift for PBMCs according to Metcalf et al. [31]. Electromagnetic simulation of the system “cell-nanostructured substrate”: (D) Numerical simulation (Δ*λ* vs Δ*T*) of the electromagnetic problem of either PBMCs (blue dots) or SW480 cancer cells (red dots) located on a nanohole structured substrate (see details in the text and Figure 2).

Before analyzing real cell samples, we checked the performance of our experimental system. We resorted to an experimental model consisting of micron-sized spherical glass particles diluted in water and deposited on the chip surface. Their RI and size parameters were provided by the commercial supplier (RI = 1.5 (soda lime glass) with diameter 10 μm). One of these particles was in the center of the illuminating spot of diameter D whose value was modified to study its influence on the effective RI of the illuminated volume and consequently, on the spectral shift undergone by the transmitted intensity. Results are shown in Figure 3A. To get the *effective fill factor* of the microsphere in the sensing volume, a plasmon penetration depth of 200 nm was assumed. From this, an estimation of the effective RI can be obtained and consequently, an estimation of the spectral shift of the transmitted intensity [21]. This shift increases as the illumination spot is reduced. This reflects the fact that the microsphere occupies a larger area of the spot. Pursuing this idea, we have applied the same methodology to PBMCs.

These cells have also a spherical shape with a diameter of about 10 μm [30] (Figure 3B), but their intrinsic optical properties are inhomogeneous due to the presence of a complex intracellular structure, including nucleus, organelles and plasma membrane in contrast to glass spheres. We have considered that surface plasmons only “see” the membrane and the surrounding cytoplasm. By following previous works on the optical properties of cells [31], we have assumed that they are homogeneous in the plasmon region with a value of their cytoplasmatic RI of 1.3572 ± 0.0002. We used a 20 μm spot to determine changes in the RI of both single microspheres and PBMCs. Results for PBMCs are shown in Figure 3C (red dots) and are compared with those of glass microspheres (blue dots). Blue dotted line marks the spectral shift of the glass microbeads obtained according to the effective RI model described before. Red dotted line marks the spectral shift for lymphocytes according to the value of their RI and reported in Metcalf et al. [31] and assuming them as geometrically spherical. The spectral positions of the analyzed PBMCs are distributed around this value with small dispersion. It is interesting to highlight that the experimental spectral positions of the measured glass beads are located below the blue dotted line corresponding to a single spherical glass bead touching the substrate according to the effective RI model described above. This means that the volume located in the plasmon range is slightly smaller than that of a bead touching the substrate. This suggests that there are Van der Waals forces between the particle and the substrate avoiding perfect contact and that these forces must be repulsive as a consequence of the different character (dielectric-metal) of the two facing bodies (microparticle-nanohole metallic substrate) [32]. These results reveal that EOT-based techniques, like the one presented here, allow us to distinguish between “microparticles” of different optical properties with high accuracy and sensitivity. Spectral wavelength shifts due to the presence of a cell (or a transparent microparticle) on a nanohole array are also accompanied by changes in the spectral transmittance (Figure 1). We have defined the parameter Δ*T* as the difference (in percentage) between the transmitted intensity with and without the cell (or a microparticle in the experimental model). We can see that Δ*T* is larger for the glass microspheres than that for PBMCs because the latter have a lower RI (similar to the buffer, ≈1.33) than the former (Figure 3C). It is important to emphasize the low dispersion in transmission shown by both microspheres and PBMCs. This is mainly due to their size and shape homogeneity. From now on, both parameters, Δ*λ* and Δ*T*, will be plotted for every case analyzed, as in Figure 3C.

### 2.3 Electromagnetic simulation of the system “cell-nanostructured substrate”

The numerical model of the problem presented in this research is just to provide a guide to interpret the experimental results and never is intended to reproduce quantitatively those results. This would result impossible for reasonable computing times by considering the current numerical tools and computing power, mainly due to the spatial optical complexity of a cell. Previous research [21] has been done on configurations like the one analyzed in this research (no cellular structure is included), cells were considered as homogeneous spheres with no structure. Their RI is evaluated by using effective RI models found in the literature [33]. Basically, Barreda et al. [21] investigated the linear response of the EOT peak shifting under variations of the effective cellular RI and the number of holes covered by the cell when it deforms (flattening effect) [21].

In this work, a more detailed structure is given to the cell and the influence of each part in the overall plasmonic response is studied. Firstly, we analyze not only the redshift but also the transmittance of the EOT maximum under variations of the cytoplasmic RI with and without a cell membrane for two non-deformed cells, corresponding to a PBMC and a HT29 colorectal cancer cell. Secondly, having fixed two cytoplasmic RI corresponding to the two types of non-deformed cells, we analyze the same two properties of the spectrum when the cell radius *r* takes a range of values. More precisely, the redshift and transmittance are studied with respect to the cell’s volume within the penetration depth. The volume of a spherical cap can be expressed in terms of the cell radius *r* as

(2) $$V=\frac{\pi r^{3}}{3}\left(2+\cos\theta \right){\left(1-\cos\theta \right)}^{2}$$

where $\theta =2\cos^{-1}\left(1-h/r\right)$ is the maximum angle subtended with respect to the centre of the sphere and *h* = 200 nm is the height of the spherical cap submerged in water and influenced by the plasmon electromagnetic field [34]. Thirdly, the effect of flattening is evaluated for two fixed volumes and RI, corresponding to PBMCs and cancer cells. This effect is geometrically simulated by considering a spherical sector whose volume is given by

(3) $$V=\frac{\pi h}{6}\left(3r_{1}^{2}+3r_{2}^{2}+h^{2}\right)$$

where h is the plasmon penetration (200 nm) and $r_{1}$ and $r_{2}$ are the two radii of the spherical sector whose values govern the flattening effect, from a hemisphere to a sphere where a spherical cap (with volume given by Eq. (2)) is immersed in the sensing volume with only a point in contact with the substrate.

The method used to keep the volume constant after modifying the flattening consisted of changing the radius of the cell until the volume of the deformed cell (computed with a tool provided by COMSOL) matched the volume of the non-deformed. All the calculations were performed with COMSOL in order to get Δ*λ* and Δ*T* for each experimental situation. The spectral transmission in the range of interest [600–800] nm, with and without the cell allows us to get the variations of *λ*_max_ and *T*_max_ (spectral position and transmission at resonance). In this range, dispersion in the optical properties of the cell components was not considered. The model was implemented with the RF module allowing us to solve the electromagnetic problem (light-matter interaction) by means of the finite element method (FEM). The discretization was constructed as a free tetrahedral distribution in terms of the excitation wavelength, guaranteeing high element density and quality for each medium (different dimensions and RI). The number of elements varied from 7 × 10^5^ to 11 × 10^5^, being the thinnest layer in the system more densified to get reliable geometrical curvatures.

To obtain a more realistic understanding of how the sensor could be used to discern between healthy and cancer cells, two samples of 50 cells with different values of cytoplasmic RI, flattening and cell radius are simulated. The membrane is supposed to present negligible dissimilarities from one cell to another, thus it is considered equal in all cases. One sample corresponds to PBMCs and the other to colorectal HT29 cell line. A Gaussian distribution of values for each parameter is considered, according to bibliographical results [35, 36] except for flattening where a uniform distribution is assumed. An example of the simulation results is shown in Figure 3D. Red dots correspond to different samples of cancer cells and blue dots to PBMCs. As Figure 3D shows, PBMCs present much lower and less dispersed transmittance changes and redshifts, because of their generally lower sizes, cytoplasmic and nuclear RIs, compared to cancer cells. According to the graph, only a restricted portion corresponds to the spectral properties of the EOT peak for PBMCs, whereas cancer cells are significantly more widespread along the map. The average redshift and transmittance for PBMCs are 1.51 and −0.044 nm, respectively, whereas for cancer cells, these values are 3.65 and −0.064 nm, respectively. Their standard deviations are 0.80 and 0.006 nm for PBMCs and 1.80 and 0.026 nm for cancer cells. As shown by the oval region representing the location for PBMCs, 17 out of the 50 cancer cells simulated laying within the dotted curve.

### 2.4 Cells

Colorectal cancer cell lines CaCo2, HT29 and SW480 were acquired from ATCC (Manassas, VA). Fibroblasts were obtained from a skin biopsy as previously described [37]. Cells were grown as monolayers; CaCo2 and fibroblasts were cultured in Dulbecco’s Modified Eagle Medium (DMEM), HT29 in McCoy’s 5A (both from Biowest, Nuaillé, France) and SW480 in Leibovitz’s L-15 medium (ATCC) each supplemented with 10% fetal bovine serum (GE Healthcare-Hyclone, Chicago, IL), 100 U/mL penicillin and 100 μg/mL streptomycin (Lonza, Basel, Switzerland) and incubated at 37 °C and 5% CO_2_ in a humidified chamber. The medium was replaced every 2–3 days. At the time of measurement, cells were detached from the culture plate with a trypsin solution (Thermo Fisher Scientific, Waltham, MA), washed with phosphate-buffered saline (PBS) (Merck, Darmstadt, Germany) and aliquoted at a density of 5 × 10^5^ cells/mL in PBS.

When indicated, forward and side scatter data of cells were collected on a FACS Canto II (BD Biosciences. La Jolla, CA).

### 2.5 Isolation of PBMCs

Peripheral blood samples (3–4 mL) were collected in EDTA-containing tubes from healthy volunteer donors. PBMCs, mainly lymphocytes (70–90%), were isolated using Ficoll-Paque density gradient according to the manufacturer’s protocol (GE Healthcare Life Sciences, Marlborough, MA) and aliquoted at a density of 3 × 10^6^ cells/mL in PBS.

### 2.6 Tissue collection and isolation of colorectal cancer cells

This study was approved by the ethics committee of Valdecilla University Hospital (Santander, Spain) and an informed written consent from healthy volunteers and patients was obtained. All research was performed in accordance with relevant guidelines and regulations.

Tumor and paired adjacent normal tissue samples were obtained from three adult colorectal cancer patients at the time of surgical resection in the Hospital Universitario Marques de Valdecilla. Tissues were analyzed by an expert pathologist. For isolation of cells, we followed a protocol previously described [36] with some modifications. Tissue samples were processed immediately after tumor resection. They were transferred to a sterile Petri dish, washed several times with PBS to remove blood cells and mechanically disaggregated by mincing using fine scissors and scalpel blades. Disaggregated tissue was incubated with 250 mg/mL Collagenase I (Sigma-Aldrich, St. Louis, MO) and 150 mg/mL DNase1 (Invitrogen, Waltham, MA) for 1 h at 37 °C on a rocking platform. Cells and remaining tissue fragments were filtered twice through 40 μm cell strainer (Falcon Brand Products, Tewksbury, MA), and centrifuged at 1700 rpm for 5 min. Cell pellet was resuspended in McCoy’s 5A supplemented with 10% fetal bovine serum and the antibiotic mix and aliquoted to a density of 5 × 10^5^/mL in PBS for analysis. The average efficacy of tumor cell enrichment (Ki67 positive) obtained after tissue processing was about 80%.

### 2.7 Plasma membrane staining

Live HT29 cells staining was performed using CellMask Green Plasma Membrane Stain (Thermo Fisher Scientific) following the manufacturer’s recommendations. Thereafter, 5 × 10^5^ cells were resuspended in 100 μL PBS and a 10 μL drop was placed onto a gold nanohole array. Confocal microscopy (Leica TCS SP5, Leica Microsystems, Wetzlar, Germany) was used to obtain images in the *xzy* plane with 40× water immersion objective. The gold array was visualized by reflection and the fluorescence signal of the cells was detected (Ex/Em = 522/535 nm).

### 2.8 Proteinase K treatment

HT29 (1 × 10^6^ cells) were incubated with 1 mg/mL Proteinase K (Sigma-Aldrich) for 1 h at 37 °C on a rocking platform and then, enzymatic activity was stopped by adding 100 μg/mL Protease Inhibitor Cocktail (Merck) in PBS containing 5 mM EDTA, for 10 min at room temperature. Cells were either fixed in 4% paraformaldehyde for immunofluorescence studiesor lysed in RIPA lysis buffer for western blot.

### 2.9 Cytoskeleton disruption and cell survival assays

CaCo2, HT29, SW480 and colorectal cancer cells (1 × 10^6^ cells) were incubated with Cytochalasin D (Sigma-Aldrich), Mycalolide B (Santa Cruz Biotechnology, Dallas, TX) or Latrunculin A (Abcam, Cambridge, UK) at a final concentration of 5 µM (dose-response study was performed) for 1 h at 37 °C and washed twice in PBS. Then, an aliquot (5 × 10^4^ cells) was used for optical measurements and the rest of the cells were fixed in 4% paraformaldehyde for immunofluorescence staining.

### 2.10 Immunopathological studies

Immunohistology was carried out on formalin-fixed 4 µm-thick paraffin-embedded tissue sections using EnVision FLEX Visualization System (Dako-Agilent Technologies, Madrid, Spain). Antibodies against the Ki67 protein (clone MIB-1) and the antigen retrieval solution-low pH were from Dako. Inmunohistochemical reactions were performed using appropriate tissue controls. Automatic staining was accomplished on a Dako Omnis autostainer (Agilent Technologies, SL).

### 2.11 Immunofluorescence

Fixed cells were first blocked in 3% bovine serum albumin (SERVA Electrophoresis GmbH, Heidelberg, Germany) for 1 h at room temperature and then, incubated with phalloidin conjugated to the fluorescent dye tetramethylrhodamine (Sigma-Aldrich) or mouse anti-human EPCAM antibody (Biolegend, San Diego, CA) for 1 h at room temperature for visualization of F-actin and cell membrane, respectively. Mouse IgG kappa binding protein conjugated to CruzFluor 488 (Santa Cruz Biotechnology) was used as secondary antibody for the mouse anti*-*human EpCAM antibody. Cells were washed in PBS and incubated with 0.2 μg/mL DAPI (Vector Lab, Burlingame, CA) for 10 min. Following centrifugation, cells were resuspended in 15 µL of ProLong Gold Antifade Reagent (Thermo Fisher Scientific) and mounted onto microscope slides. Samples were analyzed with a confocal microscope (Leica TCS SP5).

### 2.12 Western blot

Total proteins (50 µg) from cell lysates were separated onto 8% polyacrylamide gels and transferred to PVDF membranes (Thermo Fisher Scientific). Blots were blocked in 3% bovine serum albumin and incubated with antibodies against EPCAM (Biolegend) and GAPDH (Santa Cruz Biotechnology) followed by secondary anti-goat or anti-mouse antibodies conjugated to horseradish peroxidase (Santa Cruz Biotechnology). Bound antibodies were detected by a chemiluminescence assay (Thermo Fisher Scientific) in an ImageQuant LAS 4000 mini biomolecular imager (GE Healthcare).

### 2.13 Statistical analyses

Statistical sensitivity was calculated as the ratio of cells above a defined threshold to all analyzed cells, whereas specificity is the ratio of cells below a defined threshold to all analyzed cells. Two sets of 30 independent cells per sample were analyzed. Thresholds for Δ*λ* were set following the Youden index optimization criteria [38]. The p-values were calculated from a t-student test between pairs of samples, considering 30 independent normally distributed data per sample. The null hypothesis of samples with equal mean values was rejected at the p-value significance level.

## 3 Results and discussion

### 3.1 Discrimination between cancer and normal cells

**Figure 4:**
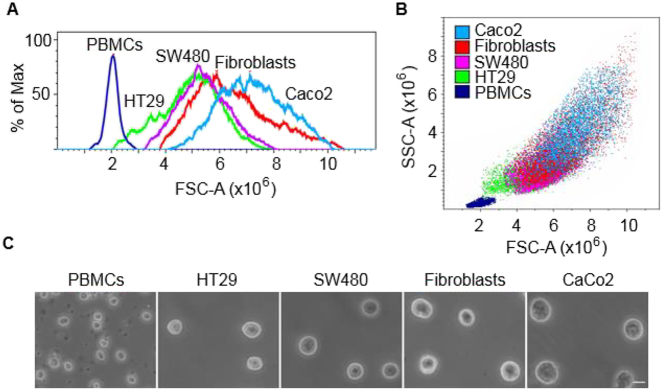
Morphological differences between cell types. (A) and (B) Flow cytometry plots showing forward (FSC) and side (SSC) scatter of colorectal cancer cells (CaCo2, SW480, HT29) and normal primary cells (skin fibroblasts, PBMCs). (C) Phase contrast microscopy images of the different cell types in suspension. Scale bar: 10 μm.

Flow cytometry is the gold standard method for characterization of cell populations at the single cell level. In the absence of fluorescent labels, flow cytometry can identify cells based on forward (size) and side (internal complexity) scatter data. However, a wide range of cancer and normal cell types have overlapping light scatter features as it happens with cells used in this study (Figure 4A–C).

**Figure 5:**
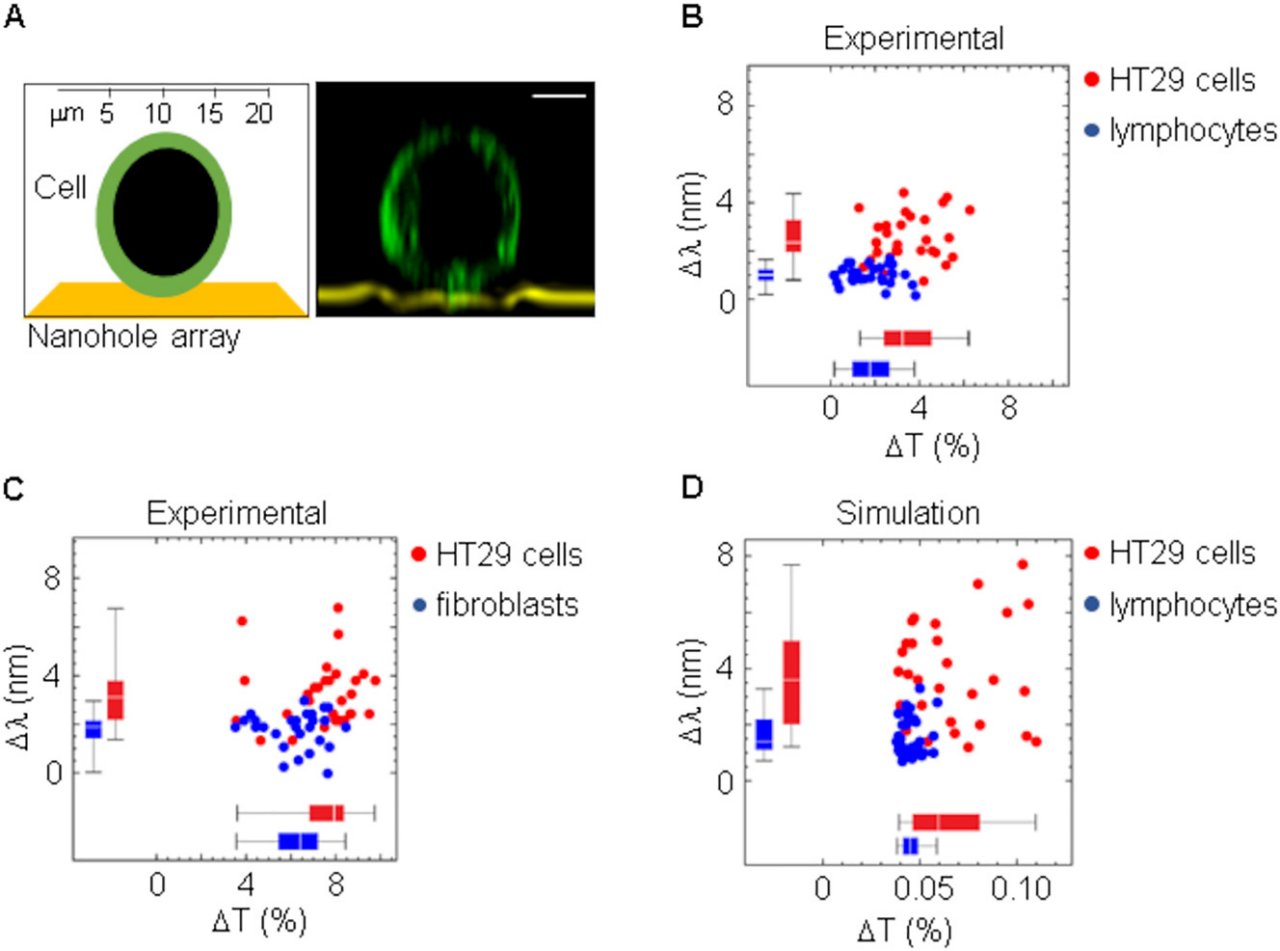
Differences in spectral shift and transmittance between HT29 colorectal cancer cells and normal primary cells. (A) Transversal confocal microscopy of a live HT29 cell on a nanohole array and a schematic representation of the image. Scale bar: 5 μm. (B) Spectral differences between HT29 cells and PBMCs. Threshold was set to 2.2 nm and sensitivity (87%) and specificity (97%) were determined. (C) Spectral differences between HT29 cells and epidermal fibroblasts. Threshold was set to 2.2 nm and sensitivity (73%) and specificity (78%) were determined. (D) Computer simulation comparing HT29 cancer cells with normal cells (PBMCs). Threshold was set to 2.7 nm and sensitivity (90%) and specificity (75%) were determined.

With our methodology, we were able to discriminate between HT29 colorectal cancer cells and normal primary cells (PBMCs and fibroblasts) (Figure 5A–C). All optical measurements were obtained in rounded cells deposited onto the sensor surface prior to cell spreading (Figure 5A). Based on our experimental results (Figure 5B), we were able to reproduce some computer simulations (Figure 5D) which intend to highlight the optical differences between normal cells (PBMCs) and cancer cells (HT29 colorectal cancer cells) (see Section 2.3 for details).

### 3.2 The discrimination capacity of the biosensor is influenced by the actin cortex

**Figure 6:**
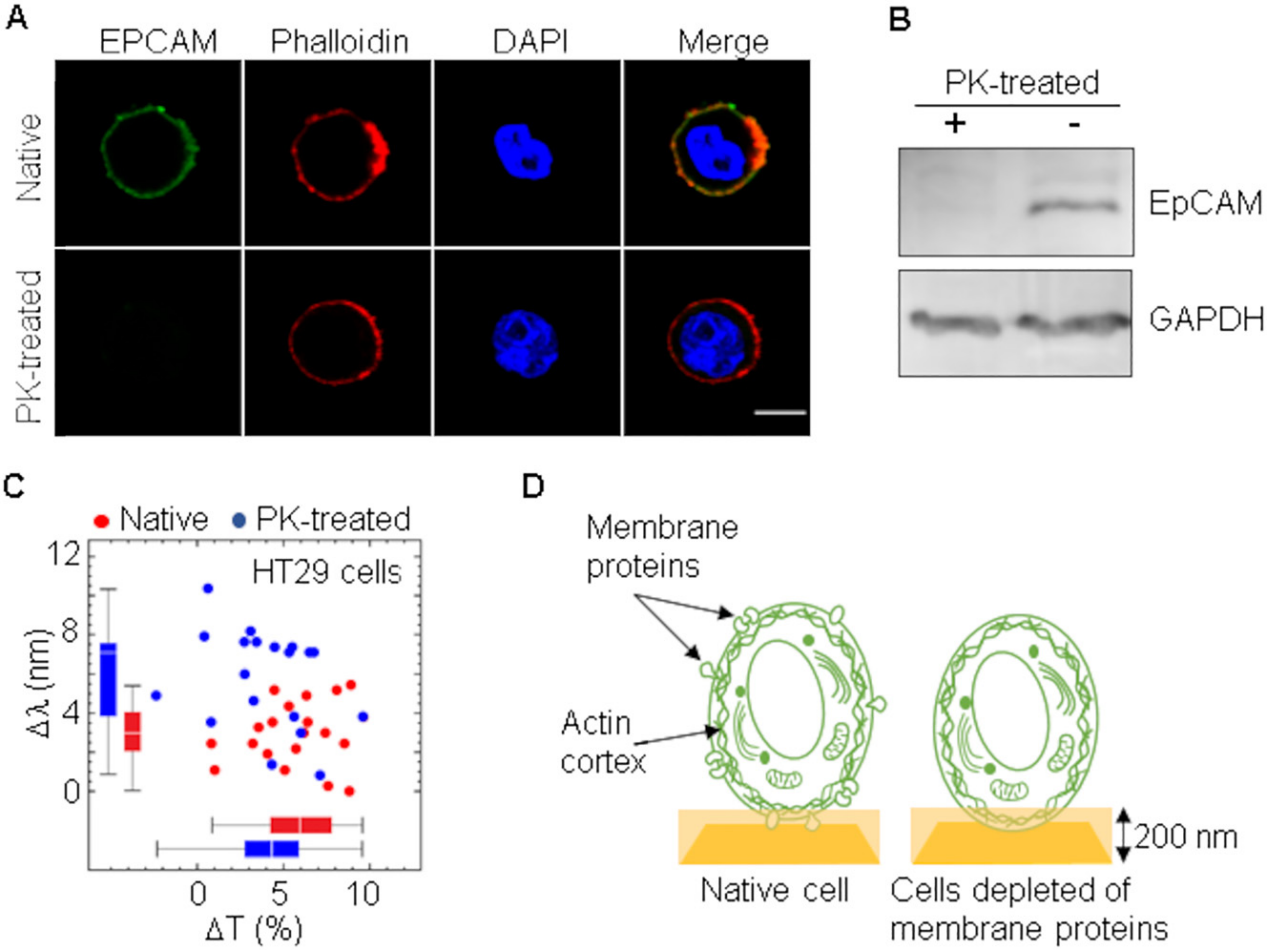
Spectral changes in cells depleted of membrane proteins. (A) HT29 cells were treated with 1 mg/mL proteinase K for 1 h at 37 °C and then fixed with 4% paraformaldehyde for immunofluorescence staining of EPCAM and actin cortex (phalloidin). DAPI was used to label nuclei. Scale bar: 5 μm. (B) Total protein was extracted from HT29 cells following incubation with proteinase K and analyzed by western blot using an anti-EPCAM antibody. Housekeeping protein GAPDH was also analyzed to assure equal loading. (C) Spectral shift and change in transmittance of cells after depletion of membrane proteins. Threshold was set to 3.8 nm and sensitivity (70%) and specificity (79%) were determined. (D) Schematic representation of a cell deposited on a nanohole array with and without membrane proteins. Plasmon depth of 200 nm is indicated.

The outer structures of the cell are proteins embedded in a lipid bilayer that form the plasma membrane, with a thickness between 4 and 10 nm [39]. These proteins have regions that extend into the space on either side of the membrane. To study the contribution of membrane proteins to the difference in RI between cells, we treated HT29 cell line with proteinase K to shave off proteins protruding into the outer space of the cell. Protease efficacy was determined by analyzing the expression of EPCAM, a membrane protein highly expressed in epithelial cancer cells [40]. Following protease treatment, EPCAM was no longer present in the surface of HT29 cells as determined by immunofluorescence (Figure 6A) and western blot (Figure 6B) analyses using anti-EPCAM antibodies.

The integrity of the cells was shown by staining the actin cortex with fluorescent phalloidin and the nucleus with DAPI (Figure 6A). Interestingly, the effective RI was higher in protease-treated cells than in native cells (Figure 6C) which indicates that membrane proteins are not essential elements for cell discrimination by EOT and that a structure in close contact with the plasma membrane could penetrate into the plasmonic field in the absence of proteins (Figure 6D). Differences between cancer cells and normal cells are based on changes in expression or mutations of particular proteins often located in the plasma membrane. However, it is estimated that membrane proteins represent a significant portion of the human proteome, constituted by far more than 20,000 proteins, 44% of which are ubiquitously expressed in all cells [41, 42]. Thus, it seems reasonable to consider that alteration of individual proteins would have a very limited, if any, effect on the organization or structure of the plasma membrane, at least to the end that could be detected by EOT. The cell cortex is a nanometer-thick layer of actin polymers arranged in filaments crosslinked into closely packed arrays by myosin proteins, underlying the plasma membrane of the cell [43, 44]. Because actin forms structures that must cover a large space, it is among the most abundant proteins in a cell. A schematic representation of the portion of the cell that could penetrate in the plasmon field generated on the surface of a nanohole array is shown in Figure 2.

**Figure 7:**
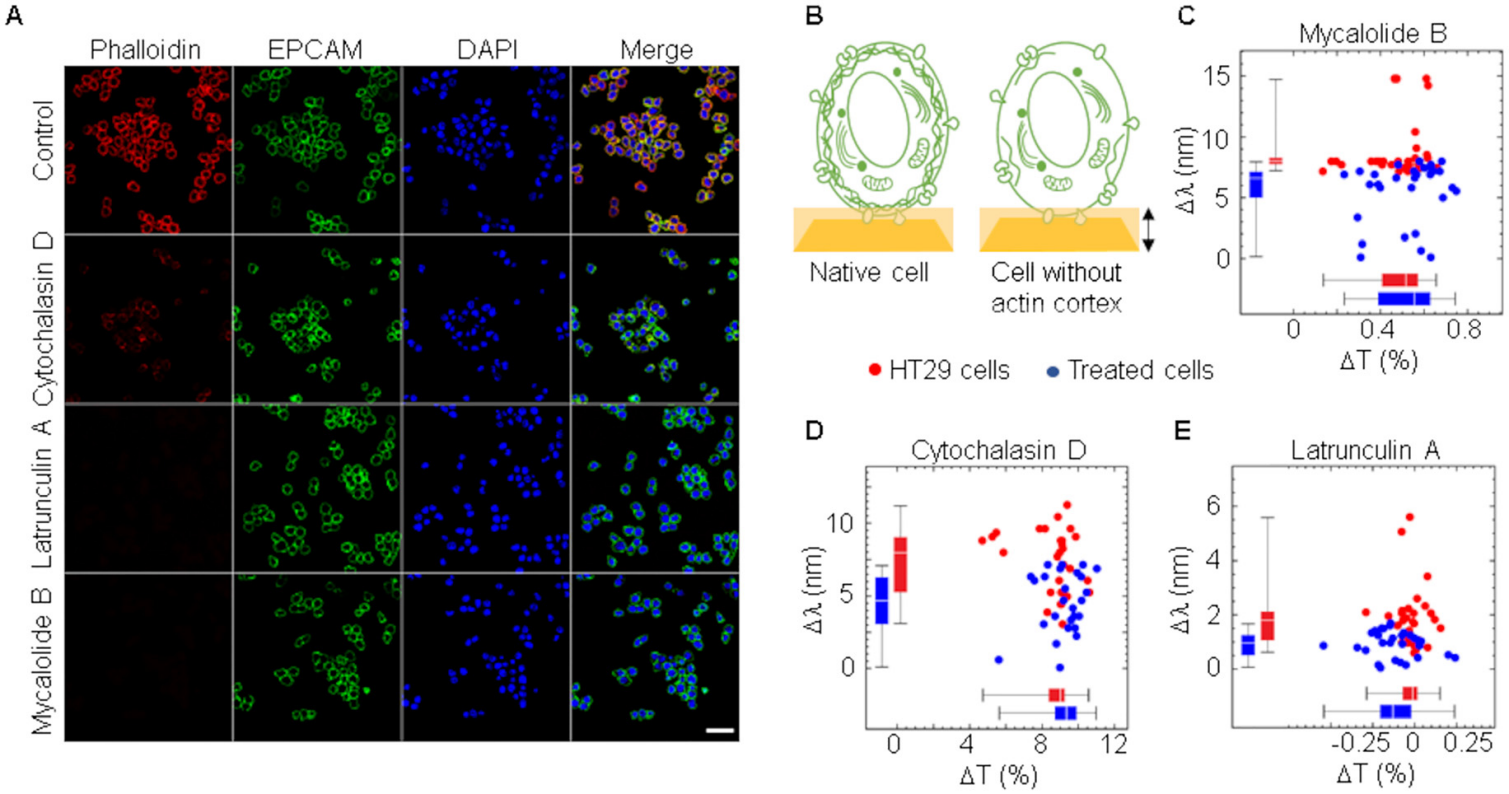
Spectral changes in cells without actin cortex. (A) HT29 cells were treated with different inhibitors of actin polymerization, Mycalolide B, Latrunculin A and Cytochalasin D at final concentration of 5 μM for 1 h at 37 °C and then fixed with 4% paraformaldehyde for immunofluorescence staining of actin cortex (phalloidin) and EPCAM. DAPI was used to label nuclei. Scale bar: 20 μm. (B) Scheme of a cell deposited on a nanohole array with and without actin cortex. Plasmon depth of 200 nm is indicated with a double arrowhead vertical arrow. (C–E) Spectral shift and change in transmittance comparing HT29 cells without and with previous incubation with F-actin inhibitors. Sensitivity and specificity of the assay were 80 and 87% (threshold set to 7.5 nm) in (C), 64 and 81% (threshold set to 6.6 nm) in (D) and 69 and 83% (threshold set to 1.4 nm) in (E), respectively.

Changes in actin cortex represent an advantage to cancer cells that become softer and facilitates cell division, infiltration and migration [45]. We treated HT29 colon cancer cells with agents that disrupt actin cortex organization either by avoiding polymerization (cytochalasin D) or promoting depolymerization (mycalolide B, latrunculin A) of actin fibers (F-actin). All of them significantly reduced the presence of F-actin as assessed by staining cells with fluorescently labeled phalloidin that binds specifically to actin fibers (Figure 7A).

**Figure 8:**
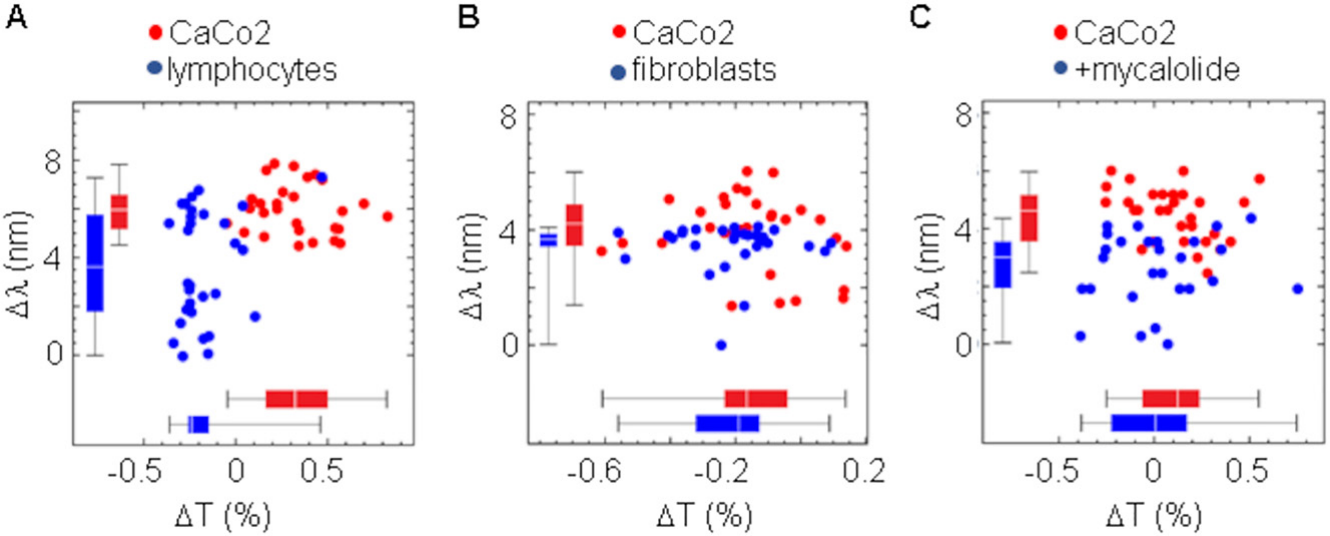
Actin cortex plays a major role in the spectral differences between CaCo2 cells and normal primary cells. (A) Spectral shift and change in transmittance comparing CaCo2 and peripheral blood lymphocytes. Threshold was set to 5.2 nm and sensitivity (71%) and specificity (60%) were determined. (B) Spectral shift and change in transmittance comparing CaCo2 and epidermal fibroblasts. Threshold was set to 4.0 nm and sensitivity (60%) and specificity (90%) were determined. (C) CaCo2 cells were incubated with actin depolarizing agent mycalolide B (5 μM for 1 h at 37 °C) and then spectral shift and change in transmittance were determined in individual cells either untreated or treated with mycalolide B. Threshold was set to 4.0 nm and sensitivity (70%) and specificity (85%) were determined.

**Figure 9:**
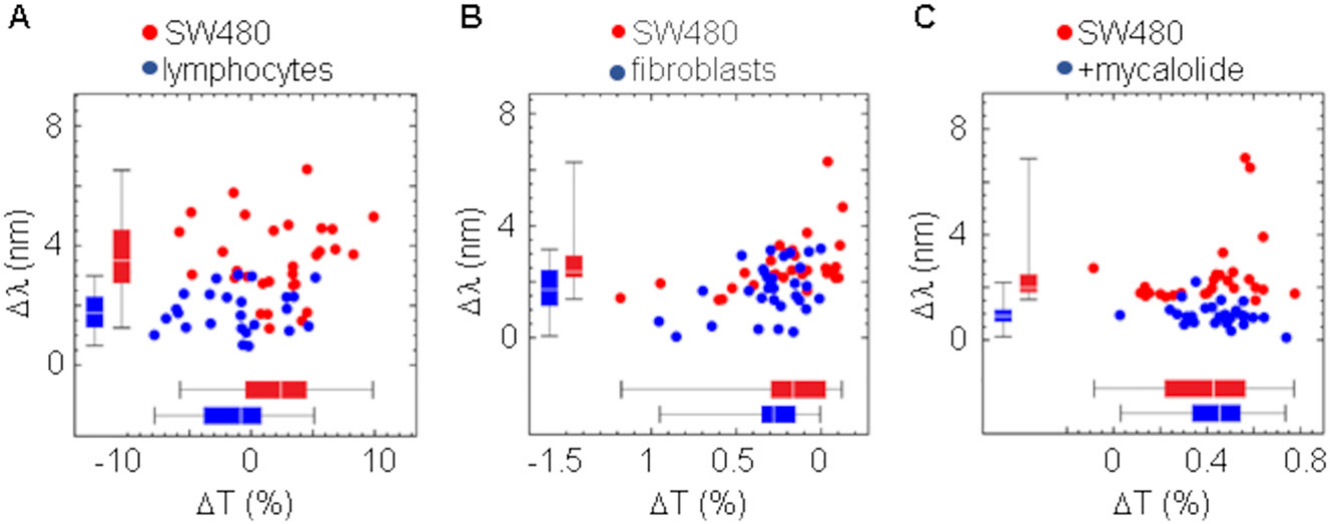
Actin cortex plays a major role in the spectral differences between SW480 cells and normal primary cells. (A) Spectral shift and change in transmittance comparing SW480 and peripheral blood lymphocytes. Threshold was set to 2.4 nm and sensitivity (83%) and specificity (84%) were determined. (B) Spectral shift and change in transmittance comparing SW480 and epidermal fibroblasts. Threshold was set to 2.2 nm and sensitivity (67%) and specificity (70%) were determined. (C) SW480 cells were incubated with actin depolarizing agent mycalolide B (5 μM for 1 h at 37 °C) and then spectral shift and change in transmittance were determined in individual cells either untreated or treated with mycalolide B. Threshold was set to 1.5 nm and sensitivity (100%) and specificity (89%) were determined.

Cell membrane labeling with anti-EPCAM antibodies revealed the integrity of the cell following treatments. The absence or reduction of a structured network of polymeric actin underneath the plasma membrane (Figure 7B) reduced the effective RI of cells under all treatment conditions (Figure 7C–E). Consistently, similar results were also obtained with two other colorectal cancer cell lines CaCo2 (Figure 8A–C) and SW480 (Figure 9A–C). In both cases, most analyzed cells showed RI values higher than PBMCs and fibroblasts, but RI was reduced following treatment with the actin depolarizing agent mycalolide B (Figures 8C and 9C). Tension forces generated by myosin reduces thickness and increases density of the actin network, leading to cell shape changes, which are key to cell migration and division, as well as cancer progression [46], [47], [48]. In line with this, changes in thickness and density of the actin cortex could help explain differences between normal and cancer cells observed by EOT. In the experimental procedure that we used to measure spectral changes, cancer cells were detached from the substrate to have individual cells in suspension acquiring a spherical morphology. However, it has been shown that cortex tension remains in cancer cells after detachment [49].

### 3.3 The biosensor discriminates between normal and tumor-derived colorectal epithelial cells

**Figure 10:**
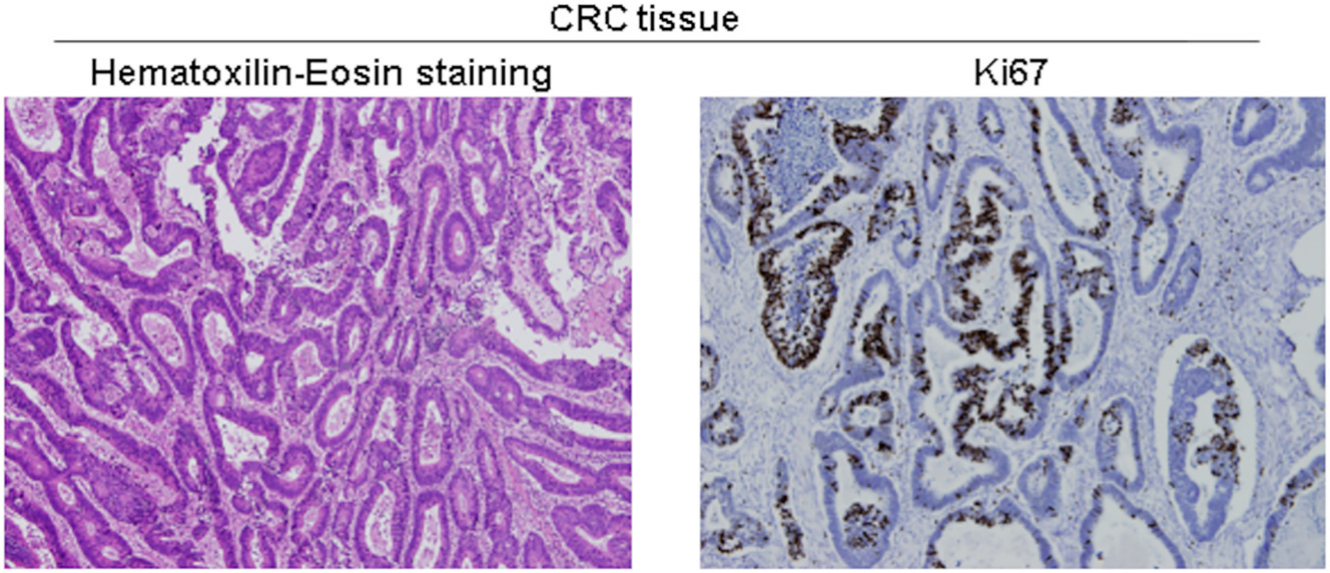
Representative hematoxilin-eosin staining of a paraffin-embedded section of colorectal cancer tissue obtained after surgery. Image shows large, hyperchromatic nuclei, characteristic of CRC cells. Tissue sections were also stained for the cell proliferation marker Ki67.

**Figure 11:**
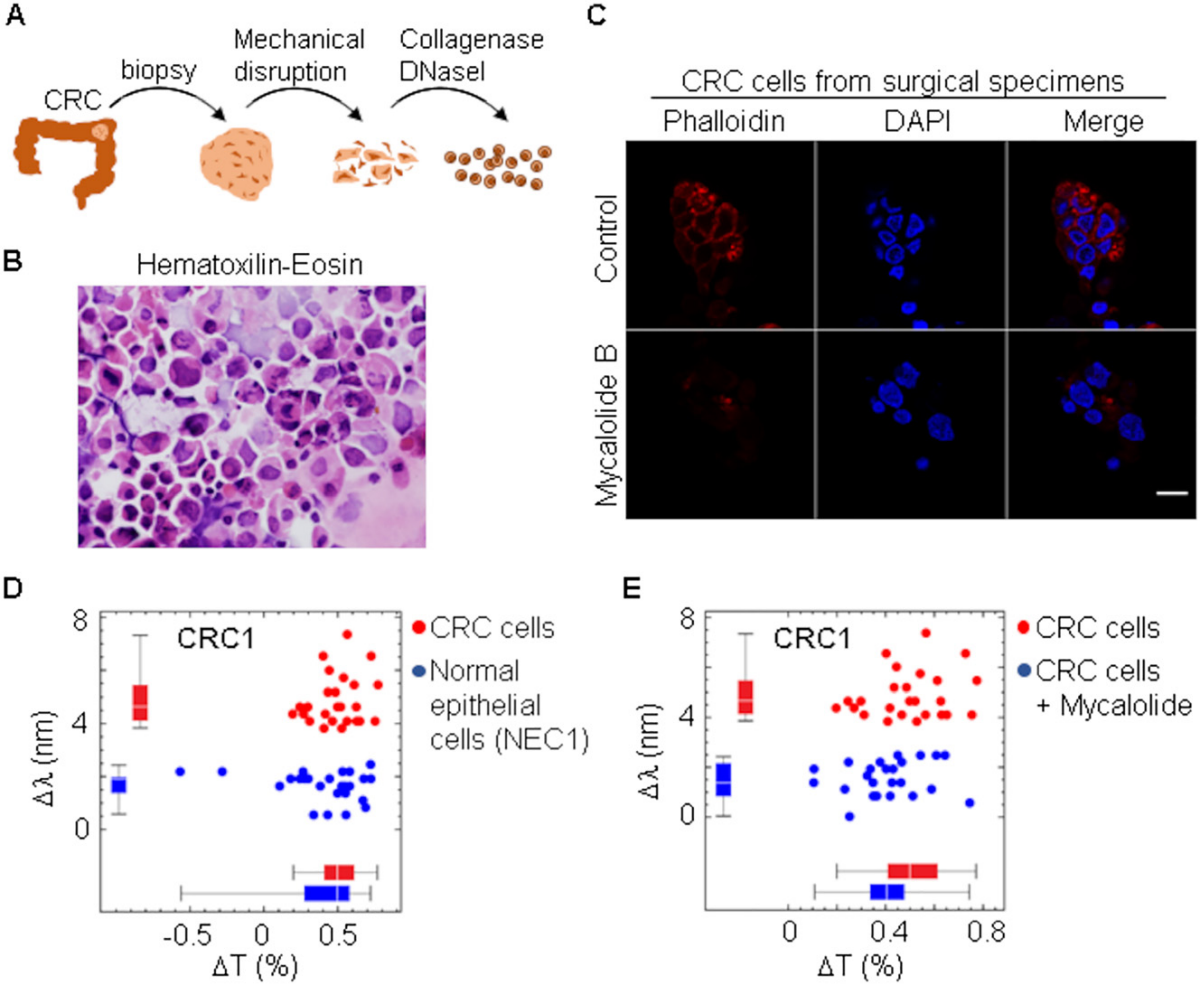
Experimental setup to study the spectral behaviour of primary cancer cells. (A) Scheme of the procedure used to obtain a population of individual cells from colorectal cancer (CRC) tissue obtained from surgical specimens. Non-necrotic tissue was washed with saline buffer and mechanically disaggregated into very small pieces. Disaggregated tissue was incubated with 250 mg/mL collagenase I and 150 mg/mL DNase I for 1 h at 37 °C and then filtered through 40 μm cell strainer to obtain individual cells. (B) Representative cytospin preparation of isolated cells after hematoxylin-eosin staining. (C) Isolated cells were fixed with 4% paraformaldehyde for immunofluorescence staining of actin cortex (phalloidin). DAPI was used to label nuclei. Scale bar: 20 μm. (D) Spectral shift and change in transmittance comparing isolated CRC cells and normal epithelial cells (NEC) obtained from the same surgical piece. (E) Spectral shift and change in transmittance comparing isolated CRC cells without and with previous treatment with 5 μM mycalolide B. Threshold was set to 3.0 nm, which determined sensitivity and specificity 100% in both (D) and (E) assays.

To further confirm our results with a model closer to primary tumors, we processed colorectal cancer tissue from surgical specimens (Figure 10) to obtain individual cells (Figure 11A, B). The same surgical piece also contained normal tissue, from which we obtained normal colon epithelial cells. As shown in Figure 11C, treatment with mycalolide B avoided or significantly reduced the formation of actin fibers.

**Figure 12:**
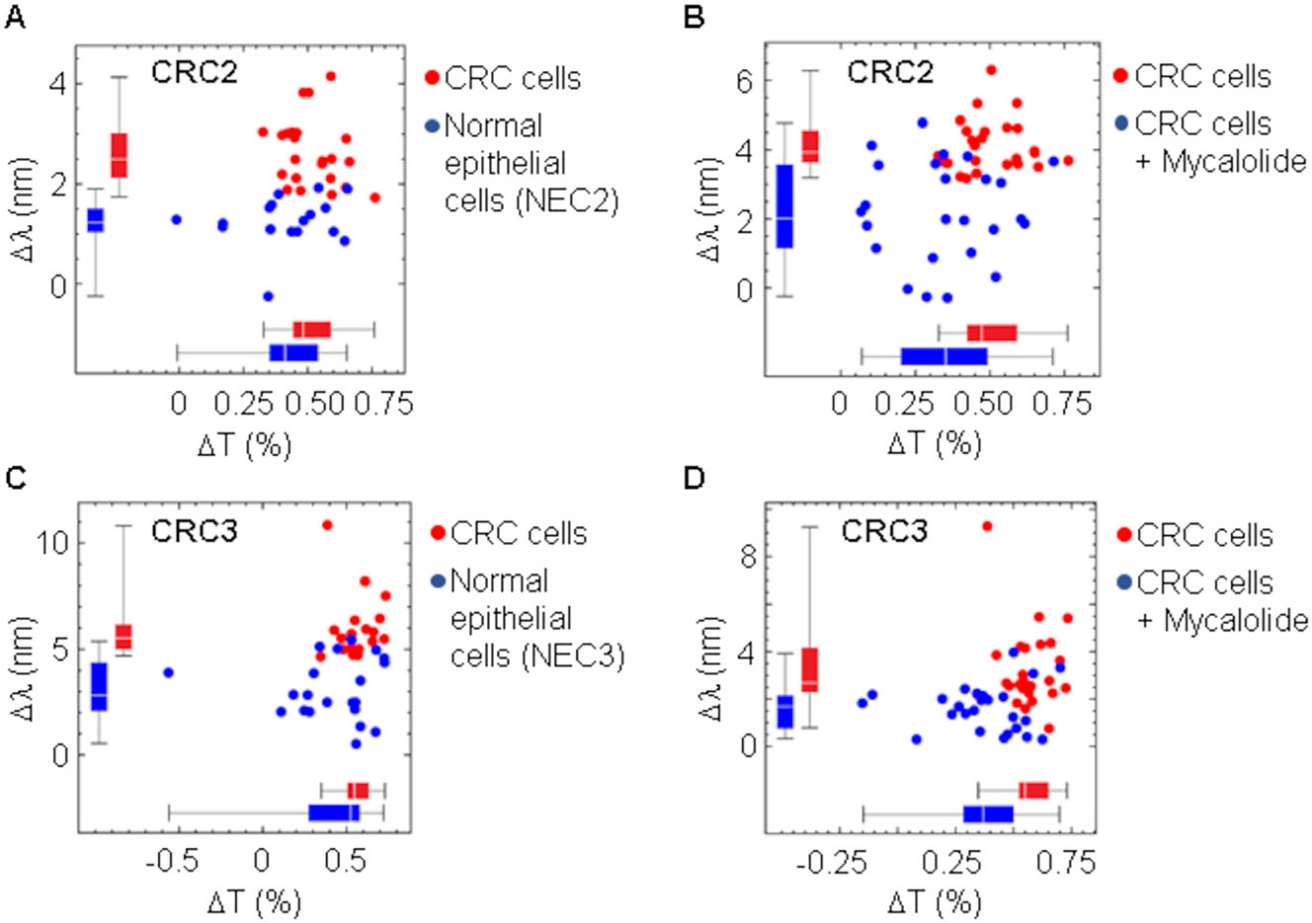
Spectral changes in CRC cells form surgical specimens. (A, C) Spectral shift and change in transmittance comparing isolated CRC cells and normal epithelial cells (NEC) obtained from two different patients, CRC2 and CRC3. (B, D) Spectral shift and change in transmittance comparing isolated CRC2 and CRC3 cells without and with previous treatment with 5 μM mycalolide B. Sensitivity and specificity of the assays were 91 and 89% (threshold set to 1.8 nm) in (A), 76 and 86% (threshold set to 3.5 nm) in (B), 80 and 100% (threshold 4.5 nm) in (C), and 72 and 81% (threshold set to 2.3 nm) in (D), respectively.

**Figure 13:**
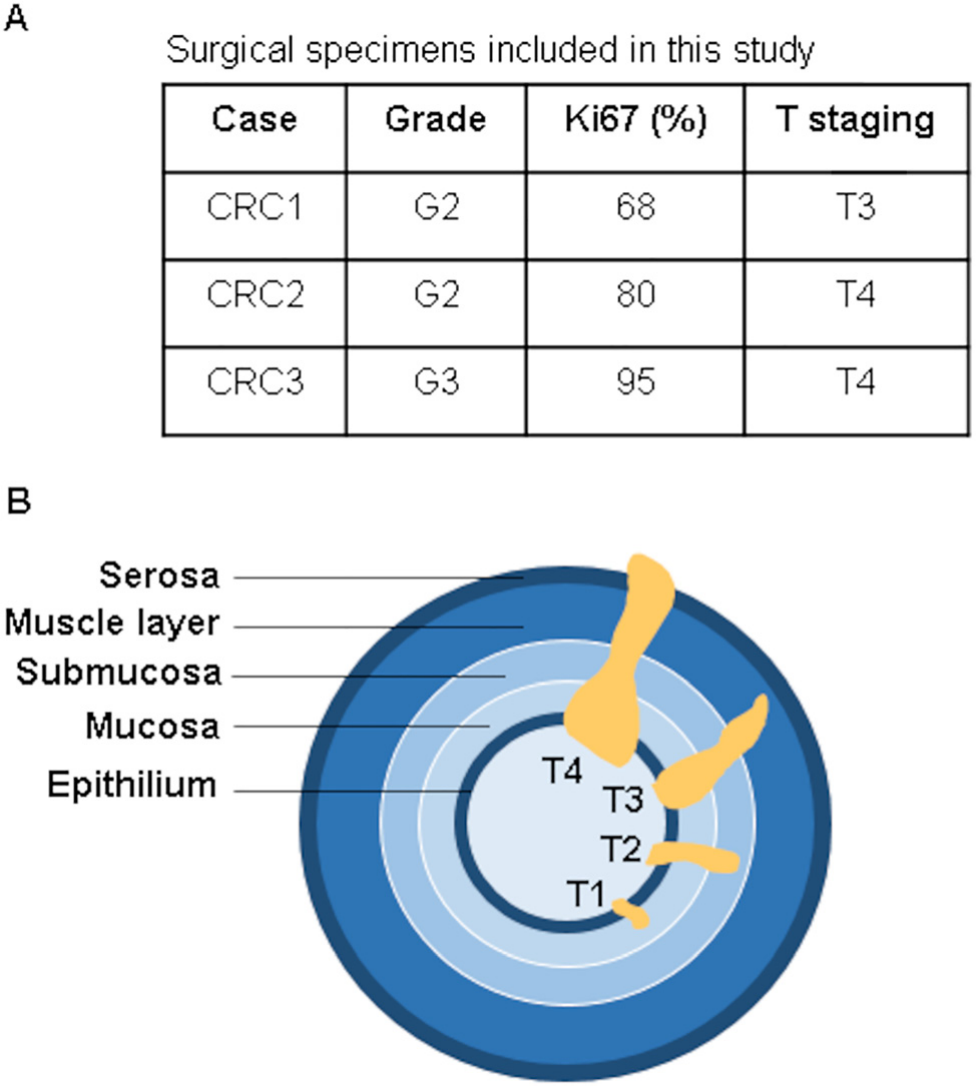
Staging and grading of colorectal cancers studied. (A) CRC1 and CRC2 are moderately differentiated (grade G2) whereas CRC3 is poorly differentiated (grade G3). In all three samples, more than 60% of cells express Ki67, which reveals a cellular state of high proliferative activity. (B) Primary tumor (T) staging classifies CRC1 as T3, invading through the muscle layer and both CRC2 and CRC3 as T4, invading through serosa and most likely reaching other organs.

Consistent with our previous results using cancer cell lines, primary tumor cells (CRC1) had an effective RI higher than normal epithelial cells (Figure 11D) and treatment with mycalolide B reduced RI to levels similar to those of normal cells (Figure 11E). To confirm these differences, we repeated the same assay with two more surgical specimens from different patients, CRC2, CRC3, and obtained similar results (Figure 12). Results with primary tumor cells raise the interesting question as to why some tumors present more clear differences in RI with respect to normal epithelia. It has been described that the contribution of actin cortex to cell elasticity is similar in normal and tumor invasive cells. However, this contribution is less relevant in tumor-noninvasive cells [25]. Of note, CRC1 sample, which displays the highest difference in RI between normal and cancer cells, is less invasive (tumor stage T3) than CRC2 and CRC3 tumors (stage T4) (Figure 13).

## 4 Conclusions

Current investigation in biomedicine, mostly cancer research, is increasingly focused on single-cell analyses to decipher tumor heterogeneity by using omics technologies [49]. In the present work, we have shown an EOT-based system that can help these technologies by discriminating between live normal and cancer cells at the single-cell level with a simple, rapid, and label-free procedure. The system performs better with primary cells from surgical specimens of colorectal cancer than with cell lines. Sensitivity and specificity of the assay for discriminating normal colon epithelial cells from colorectal cancer cells are 80–100% and 89–100%, respectively. Although the experiments have been performed with a single configuration of a nanoarray-based sensor chip, other configurations changing the nanostructured surface might contribute to increase the specificity of the system. To achieve this, and to prove the utility of the system in the analysis of other types of cancer cells, availability of a higher number of surgical specimens would be needed. Our detection system could be easily adapted to single-cell isolation devices such as a micro-pipetting cell picker, successfully used to isolate single cells for transcriptomic analyses [50, 51]. Differences in EOT are greatly influenced by the actin filament network that forms the cell cortex, a structure that is essential for cancer cells to divide and invade the surrounding tissue. However, we cannot rule out that other biological structures in close proximity to the inner plasma membrane, including proteins that interact with actin filaments may also contribute to cell discrimination capacity of the system. Our findings open the possibility to explore processes that depend on the actin cytoskeleton dynamics, including different stages of cancer cells such as differentiation, proliferation, and invasiveness. Additionally, proper functioning of the immune system, a key player to prevent and fight cancer, requires immune cells to be able to migrate into tissues, establish cell-cell interactions and activates endocytosis, which rely on the dynamics of actin cytoskeleton [52]. Thus, EOT could help analyzing tumor staging and the immune system activation status in cancer patients at the single-cell level. Further studies including multiple surgical samples from cancers with different stages will boost the potential biomedical applications of this plasmonic system.
